# Losartan Alleviates Renal Fibrosis and Inhibits Endothelial-to-Mesenchymal Transition (EMT) Under High-Fat Diet-Induced Hyperglycemia

**DOI:** 10.3389/fphar.2018.01213

**Published:** 2018-10-29

**Authors:** Yufeng Yao, Yong Li, Xiaofei Zeng, Zheng Ye, Xia Li, Lu Zhang

**Affiliations:** ^1^Key Laboratory of Molecular Biophysics of the Ministry of Education, College of Life Science and Technology and Center for Human Genome Research, Huazhong University of Science and Technology, Wuhan, China; ^2^College of Life Sciences, Wuhan University, Wuhan, China; ^3^College of Biological Science and Medical Engineering, Southeast University, Nanjing, China; ^4^Institute of Agricultural Resources and Environment, Guangdong Academy of Agricultural Sciences, Guangdong, China; ^5^Wenhua College, Huazhong University of Science and Technology, Wuhan, China

**Keywords:** EMT, diabetic nephropathy, TGF-β1/Smad signaling, losartan, oxidative stress

## Abstract

The endothelial-to-mesenchymal transition (EMT) of glomerular vascular endothelial cells is considered to be pivotal in diabetic nephropathy (DN). The risk of DN can be decreased by losartan, but the potential molecular mechanism(s) are not fully understood. Extensive data show that the EMT occurs in proximal tubular endothelial cells resulting in an endothelial phenotype switch (fibrotic matrix accumulation), consequently enhancing the development of renal interstitial fibrosis. Here, we found that losartan significantly ameliorated DN-induced renal fibrosis progression via inhibition of the EMT in mice. *In vivo* experiments suggested that losartan significantly alleviated microalbuminuria and pathologic changes under high-fat diet-induced hyperglycemia. Immunohistochemistry indicated that losartan suppressed the EMT in glomeruli. In addition, losartan decreased oxidative stress damage and inhibited the transforming growth factor (TGF)-β1/Smad pathway. Furthermore, consistent changes were detected *in vitro* where losartan markedly inhibited the EMT and TGF-β1/Smad pathway induced by high glucose in glomerular endothelial cells. Together, these results suggested that losartan could alleviate the EMT in glomeruli via inhibition of oxidative stress damage and the TGF-β1/Smad signaling pathway under hyperglycemia.

## Introduction

Diabetes mellitus is characterized by hyperglycemia caused by the impairment of insulin secretion, insulin resistance (IR), or both (Wild et al., 2004; Tsai et al., 2018). Excessive caloric intake (due to unhealthy lifestyle) increases the risk of development of type 2 diabetes mellitus (T2DM) (Mourad et al., 2013). High-fat diet (HFD) intake results in progressive lipid accumulation in liver and muscles that conducting to activation of inflammatory cytokines and IR (Brownlee, 2005; Mourad et al., 2013). In T2DM, β-cell failure (a loss of insulin-producing β-cells) results from a multifactorial process initiated by IR, often in the setting of obesity (Weir and Bonner-Weir, 2004; Alejandro et al., 2015). The consequence of obesity is characterized by IR, hyperglycemia, atherosclerosis, dyslipidemia, and hypertension. As reported, IR in T2DM and metabolic stress (mainly hyperglycemia and hyperlipidemia) acts as major risk factor for diabetic diseases (Bai et al., 2016). These risk factors referred to as metabolic syndrome (Decleves et al., 2013). Metabolic syndrome is known to be associated with a dysregulation of catalytic activities or expression levels of the metabolic enzymes that play a pivotal role in synthesis and/or consumption of glucose, lipid, and proteins (Wei et al., 2018).

Diabetic nephropathy (DN) is the most common complication of patients with diabetes. In addition, DN causes progressive renal fibrosis that finally results in end-stage renal disease (ESRD) (Komorowsky et al., 2012; Park, 2014; Ma et al., 2017; Geng et al., 2018). Clinical studies find that glomerular basement membrane thickening, extracellular matrix accumulation, glomerular sclerosis, renal tubular atrophy, and renal interstitial fibrosis are pathological characteristics of DN (Dormandy et al., 2005; Kolset et al., 2012). Many factors contribute to the development of DN such as oxidative stress, chemokines, and inflammation (Donate-Correa et al., 2015; Bhattacharjee et al., 2016; Barman et al., 2018). Studies show that the DN pathological process is irreversible and resistant to clinical therapy (Ma et al., 2017). Therefore, it is necessary to identify novel therapeutic drugs to delay the progression of DN.

Microalbuminuria and hyperfiltration in the glomerulus of diabetic kidneys are early features of DN (Fu et al., 2015; Qi et al., 2017; Daehn, 2018). Glomerular vascular endothelial cells (GVECs) are considered to be critical in maintaining the glomerular filtration barrier (Satchell and Tooke, 2008; Satchell, 2012; Fu et al., 2015). Injury to GVECs may lead to filtration of albumin and renal fibrosis (Ma et al., 2017). Recent studies indicate that the endothelial-to-mesenchymal transition (EMT) is a potential source of activated fibroblasts that finally result in renal fibrosis (Peng et al., 2016; Zhao et al., 2016; Daehn, 2018).

Hyperglycemia can induce proinflammatory factors and activate the profibrotic EMT of GVECs in kidneys (Peng et al., 2016; Yu et al., 2017). Once the EMT is activated, endothelial cells down-regulate the expression of endothelial phenotype-specific markers, such as CD31 and VE-cadherin, while mesenchymal feature-specific markers, such as α-smooth muscle actin (α-SMA) and vimentin, are up-regulated (Cruz-Solbes and Youker, 2017; Shang et al., 2017; Yu et al., 2017). Consequently, the EMT of these cells leads to activated fibroblasts and enhances the pathological process of renal fibrosis.

Recent data have demonstrated that the transforming growth factor (TGF)-β1/Smad pathway is crucial in the EMT (Wynn, 2008; Xu et al., 2018). High glucose (HG) could increase the protein level of TGF-β1, which is a strong stimulus of the EMT in GVECs (Li et al., 2015; Zhang et al., 2018). Losartan is a classic selective angiotensin II receptor antagonist (Su et al., 2018). The antifibrotic effect of losartan has been elucidated in numerous fibrotic processes (Yu et al., 2002; Wengrower et al., 2012; Guo et al., 2015; Salama et al., 2016). Furthermore, losartan can significantly blunt the TGF-β1/Smad signaling pathway in many different tissues (Bar-Klein et al., 2014; Wu et al., 2016). In this study, we elucidated whether losartan could alleviate renal fibrosis under hyperglycemia in glomeruli through inhibition of the EMT and examined the potential molecular mechanisms.

## Materials and Methods

### Mice Model and Related Protocols

All experimental procedures involving animals were approved by the Ethics Committee of College of Life Science and Technology at Huazhong University of Science and Technology. C57BL/6J mice (male, 6–8 weeks) were purchased from Center for Medical Experimental Animals of Wuhan University (Wuhan, China). All mice were maintained in cages (4–6 mice/cage) under a standard light/dark cycle (12:12/h). Male C57BL/6J mice were fed with HFD (60% fat, 20% protein, and 20% carbohydrate as percentages of total kcal) to create a model for hyperglycemia. Briefly, C57BL/6J mice were divided into to a standard diet (STD) group or a HFD group randomly for 30 weeks and then administrated either with losartan or a placebo for an additional 6 weeks. Blood glucose levels were measured with a glucometer (SANNUO, Changsha, China). Mice were divided into three groups randomly: STD control group (*n* = 12); (2) HFD group (diabetic group, *n* = 12); (3) HFD + losartan group (losartan-treated diabetic group, *n* = 12), mice were treated with oral losartan (20 mg/kg/day) in distilled water. Mice in STD and HFD groups were administered y with an equal volume of distilled water, respectively (Decleves et al., 2013).

### Cell Culture and Treatments

Human renal glomerular endothelial cells (GEnCs) were purchased from Sciencell (Carlsbad, CA, United States) and maintained in endothelial cell medium (ECM) supplemented with 10% (volume/volume) fetal bovine serum (Gibico Life Technologies, MD, United States) and 1% endothelial cell growth supplement in a humidified water jacket incubator (Thermo Fisher Scientific, MA, United States) with 5% CO_2_ at 37°C. For losartan related *in vitro* experiments, GEnCs were divided into three groups: (1) NG group (negative control group, L-glucose), (2) HG group (high D-glucose group, 30 mmol/L), (3) HG + Los group (high glucose + losartan). For cells losartan treatment, a dose of 20 μM was used. After exposure to glucose for 48 h, cells were harvested for cell lysates preparation and subject to further analysis.

### Immunohistochemistry Staining

In brief, the mice were anesthetized with an intraperitoneal injection of sodium pentobarbital (50 mg/kg). Kidneys were harvested and then fixed overnight in 4% paraformaldehyde (PFA), embedded in paraffin, and sectioned (4.5 μm). Embedded sections were deparaffinized, dehydrated, and rehydrated after being sectioned. After microwave antigen retrieval, endogenous peroxidase blocking at room temperature for 15 mins in dark and normal goat serum blocking in equilibration buffer at room temperature (50 μl/section), sections were subject to immunohistochemical analysis using a polyclonal antibody against CD31, CD68, Mcp1, α-SMA. 3, 30-diaminobenzidine (DAB) was used as a chromogenic substrate, and the sections were counterstained with hematoxylin. Images were photographed with an inverted Nikon Eclipse Ti microscope (Nikon, Tokyo, Japan). Images were further analyzed with the Image-Pro Plus version 6.0 software (Media Cybernetics Inc., MD, United States). For Masson staining, briefly, kidney samples were fixed, sectioned, deparaffinized, stained with Masson composite staining solution, washed with 0.2% acetic acid solution, 5% phosphotungstic acid solution, 0.2% acetic acid solution. After stained with bright green staining solution, washed twice with 0.2% acetic acid solution, dehydrated in absolute alcohol, put in xylene for transparency, and finally sealed with neutral gum for further analysis (Lu et al., 2016; Yao et al., 2017a,b).

### Isolation of Mouse Renal Glomeruli Endothelial Cells (MRGECs)

Mice were anesthetized with an intraperitoneal injection of sodium pentobarbital (50 mg/kg). Kidneys were decapsulated and then cut the cortex away from the medulla, chop the cortex into 1–2 mm^2^ pieces. Press this preparation through a sieve of mesh size 250 μm, into a sterile Petri dish on ice, using a 5-mL syringe barrel. This results in the separation of glomeruli from renal tubules, interstitium, and vasculature. Transfer the glomerular-enriched filtrate from the Petri dish into sterile 50 mL falcon tubes on ice. Further pressed paste-like preparation gently through a 105-μm sieve and a second 75-μm filter, washed extensively with PBS. Retained glomeruli were retrieved into 5 mL PBS, washed another three times with PBS and interval centrifugation (2000 r/min, 5 min). Resuspend enriched glomeruli in collagenase solution and incubate at 37°C for 20 mins, collect pellets after centrifugation, and further transferred to gelatin-coated cell culture flask. After 2 weeks of culture, adhered cells were subject to Mini MACS magenetic cells (CD34^+^) separation for MRGECs according the manufacturers instruction.

### Pathological Analysis for Glomerular Volume (GV)

In brief, the mean glomerular volume was calculated from the measured GA (glomerular area) as follows: GV = (GA)3/2 × β/d, where β is a dimensionless shape coefficient (β = 1.38 for spheres), and *d* is a size distribution coefficient (*d* = 1.01). GA was defined as the area described by the outer capillary loops of the tuft using a computer imaging analyzer (Image J), *d* was used to adjust for variations in glomerular size (Awazu et al., 2003; Kobayashi et al., 2015).

### Real-Time Quantitative PCR

In brief, total RNA was isolated from cells using Trizol reagent (TaKaRa, Dalian, China) and then subject to cDNA synthesis with M-MLV reverse transcriptase (Promega, WI, United States). DNase I (Promega, WI, United States) was used to remove genomic DNA contamination before reverse transcription. Quantitative PCR analysis was carried out using the FastStart Universal SYBR Green Master (Roche, Basel, Switzerland) and a 7900 HT Fast Real-Time PCR System (ABI, IL, United States) (Zhang et al., 2016).

### Western Blotting

Glomerular endothelial cells (GEnCs) lysates were prepared using Western-IP lysis buffer (Beyotime, Beijing, China) supplemented with proteinase inhibitor cocktail (Roche, Basel, Switzerland). Total protein extracts were subjected to targeting proteins separating, transferred to a PVDF membrane, and blotted with antibodies against targeting proteins (collagen I, collagen III, fibronectin, CD31, α-SMA, VE-cadherin, vimentin, NOX-4, TGF-β1, p-Smad2, t-Smad2, p-Smad3, t-Smad3, GAPDH). The secondary antibody was a goat HRP-conjugated secondary antibody (1:20,000 dilution). The images were captured using a ChemiDoc XRS (Bio-Rad Laboratories, CA, United States) with the SuperSignal West Pico Chemiluminescent Substrate (Pierce Chemical Co., IL, United States) and further analyzed with Gel-Pro analyzer (Ye et al., 2016).

### Statistical Analysis

We have repeated all experiments at least three times. The data were presented as mean ± SD. Statistical analysis was performed using the Student’s *t-*test. *P*-value < 0.05 was considered to be statistically significant.

## Results

### Effects of Losartan on Kidney Hypertrophy Under High-Fat Diet-induced Hyperglycemia

**FIGURE 1:**
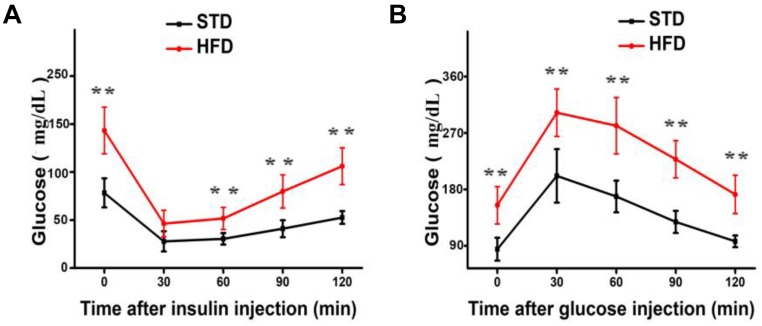
Long-term HFD induces insulin resistance and glucose tolerance. **(A)** Insulin resistance assay was performed on mice after 30 weeks of a HFD (*n* = 12/group). **(B)** Glucose tolerance assay was performed on mice after 30 weeks of a HFD (*n* = 6 for each group). Data are shown as mean ± SD. ^∗∗^*P* < 0.01.

It is well established that a long-term HFD induces IR and glucose intolerance. Indeed, HFD-fed mice showed impaired IR and glucose tolerance sensitivity compared to STD-fed mice (Figures 1A,B). These results indicate that hyperglycemia model was successfully induced by HFD.

**FIGURE 2:**
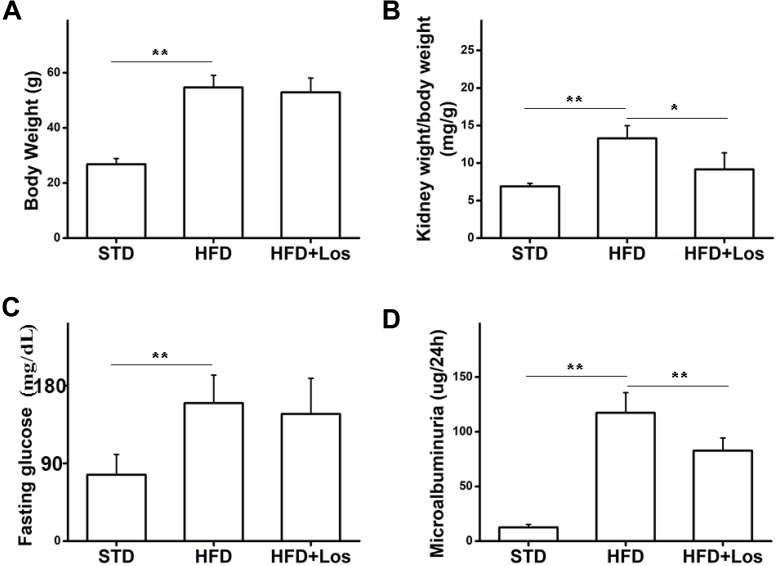
Effects of losartan on metabolic parameters in diabetic mice. **(A)** Effect of losartan on body weight (BW). **(B)** Effect of losartan on kidney weight/body weight (KW/BW). **(C)** Fasting glucose evaluation of mice from STD group, HFD group, and HFD + losartan group. **(D)** Microalbuminuria measurement of mice from STD group, HFD group and HFD + losartan group, *n* = 12/group. Three independent experiments were performed. Data are shown as mean ± SD. ^∗^*P* < 0.05, ^∗∗^*P* < 0.01.

To study the role of losartan in glucose metabolism and obesity, we first determined the effect of losartan on body weight. Our data showed that the body weights of mice from the HFD and HFD + losartan groups were increased significantly compared with the STD group. No statistical change was found between the HFD and losartan-treated HFD groups (Figure 2A).

Hyperglycemia was successfully induced by the HFD (60% fat, 20% protein, and 20% carbohydrate as percentages of total kcal; SaiNuoBio, Beijing, China) for up to 30 weeks (Figure 2C). Furthermore, microalbuminuria (a key functional parameter in DN) was significantly increased in HFD compared with STD mice, and losartan administration mediated a partial recovery (Figure 2D). Glomerular hypertrophy was generally evaluated using the KW/BW ratio. Our data showed that KW/BW ratio of HFD mice was significantly greater than STD mice, while KW/BW was much reduced in the HFD + losartan group (Figure 2B). These data suggest that losartan effectively attenuates kidney hypertrophy.

### Losartan Inhibits Hyperglycemia Induced Glomeruli Fibrosis and Inflammation

**FIGURE 3:**
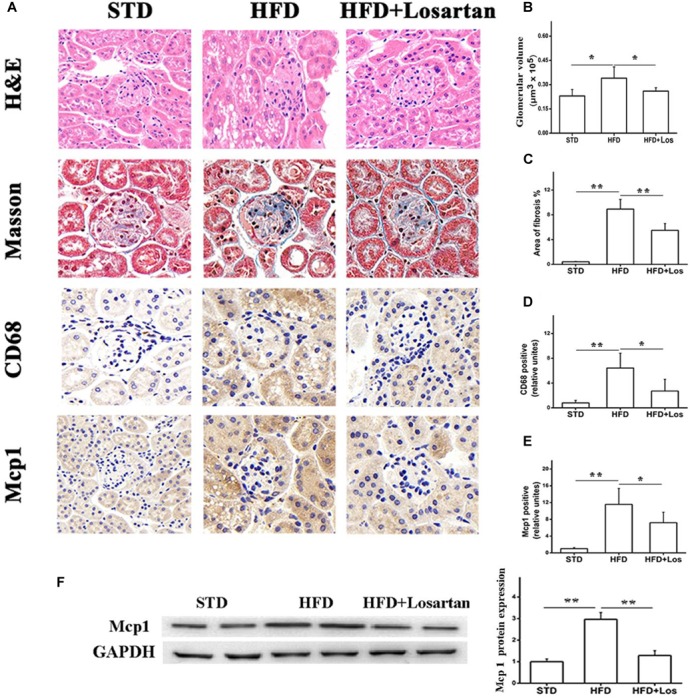
Pathological analysis of diabetic mice kidney tissues. **(A)** H&E staining, Masson staining, and immunostaining analysis of inflammation markers (CD68, Mcp1) in different groups. **(B)** Glomerular volume analysis of kidney tissues. **(C)** Fibrosis quantification of mice kidneys from different groups. **(D,E)** Inflammation quantification of kidney tissues from different groups, *n* = 12/group. **(F)** Kidney tissue content of MCP-1 analysis. Three independent experiments were performed. Data are shown as mean ± SD. ^∗^*P* < 0.05, ^∗∗^*P* < 0.01.

H&E staining analysis showed that HFD mice developed a larger glomerular volume compared with STD mice. However, this change was impaired by administration of losartan (Figures 3A first row, B). For assessing fibrosis, Masson trichrome staining indicated the interstitial fibrosis (blue) area. Fibrosis was markedly increased in HFD compared with STD mice, while it was significantly decreased by losartan treatment (Figures 3A second row, C). In addition, immunohistochemistry of inflammation markers (CD68 and Mcp1) suggested that the inflammation process was also enhanced in HFD compared with STD mice, while losartan treatment significantly attenuated this change (Figures 3A third row, D,E). We got similar results in kidney tissues for Mcp1 protein level using Western blotting assay (Figure 3F). Quantification of fibrosis and inflammation is shown in Figures 3C–E.

**FIGURE 4:**
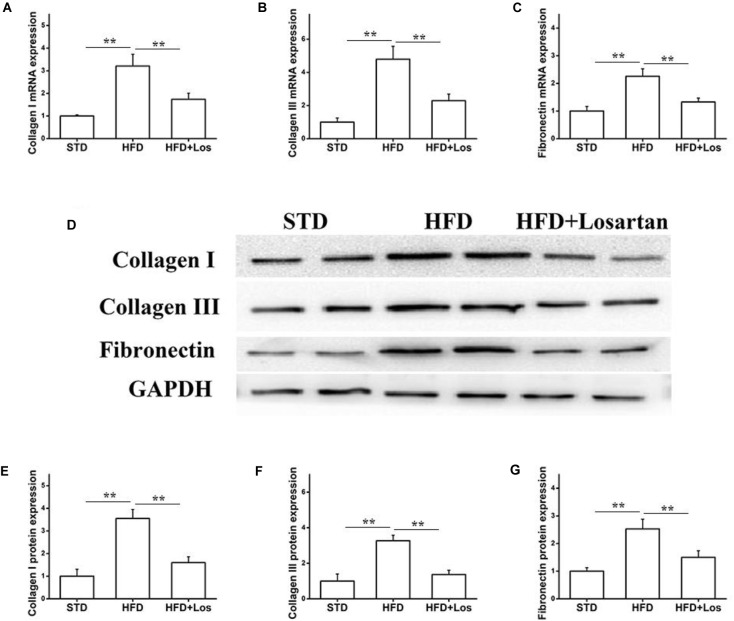
Effect of losartan on fibrosis in kidneys. **(A–C)** The mRNA levels of *collagen I*, *collagen III*, and *fibronectin* in kidneys. **(D)** Western blotting assay of collagen I, collagen III and fibronectin in different groups. **(E–G)** Quantification of collagen I, collagen III, and fibronectin for Western blotting in different groups, GAPDH was used as a loading control. Three independent experiments were performed. Data are shown as mean ± SD. ^∗∗^*P* < 0.01.

We also carried out real time qPCR and Western blotting analysis to evaluate the fibrosis status of kidneys. The mRNA levels of collagen I, collagen III, and fibronectin were significantly increased in HFD compared with STD mice, and markedly decreased by losartan administration (Figures 4A–C). Similarly, the kidneys protein levels of collagen I, collagen III, and fibronectin were significantly increased in HFD compared with STD mice, while they were markedly decreased by losartan (Figure 4D). Quantification of collagen I, collagen III, and fibronectin expression is shown in Figures 4E–G. These data suggest that losartan functions as a protective reagent against fibrosis and inflammation under hyperglycemia.

### Losartan Alleviates the EMT in Glomeruli Under Hyperglycemia

**FIGURE 5:**
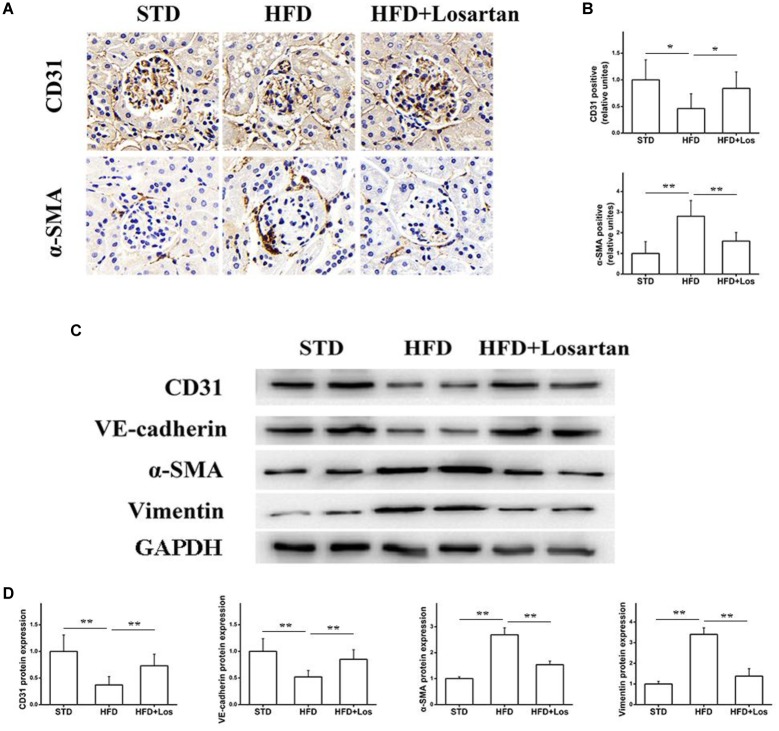
Effect of losartan on EndMT in kidneys. **(A,B)** Immunostaining for CD31 and α-SMA. **(C)** Western blotting assay of endothelial cell markers (CD31, VE-cadherin) and fibroblast markers (α-SMA, vimentin) in different groups. **(D)** Quantification of CD31, VE-cadherin, α-SMA, and vimentin for Western blotting assay in different groups, GAPDH was used as a loading control, *n* = 12/group. Three independent experiments were performed. Data are shown as mean ± SD. ^∗^*P* < 0.05, ^∗∗^*P* < 0.01.

The EMT is a process in which endothelial cells lose their endothelial features (CD31 and VE-cadherin) and gain mesenchymal features (vimentin and α-SMA) (Su et al., 2018). To assess the effect of losartan on the EMT in glomeruli under hyperglycemia, immunohistochemistry was performed. The results showed that the protein level of CD31 in glomeruli was markedly reduced, but the protein level of α-SMA was increased significantly in HFD compared with STD mice. Furthermore, these changes were impaired by the administration of losartan (Figures 5A,B). Western blotting assays indicated that both CD31 and VE-cadherin were decreased significantly in HFD compared with STD mice, and losartan administration mediated a partial recovery. In contrast, the protein levels of mesenchymal markers (α-SMA and vimentin) were increased in HFD compared with STD mice, while these changes were reduced by losartan administration (Figures 5C,D). These data suggest that losartan alleviates the EMT under hyperglycemia.

### Effects of Losartan Administration on Oxidative Stress Under Hyperglycemia

**FIGURE 6:**
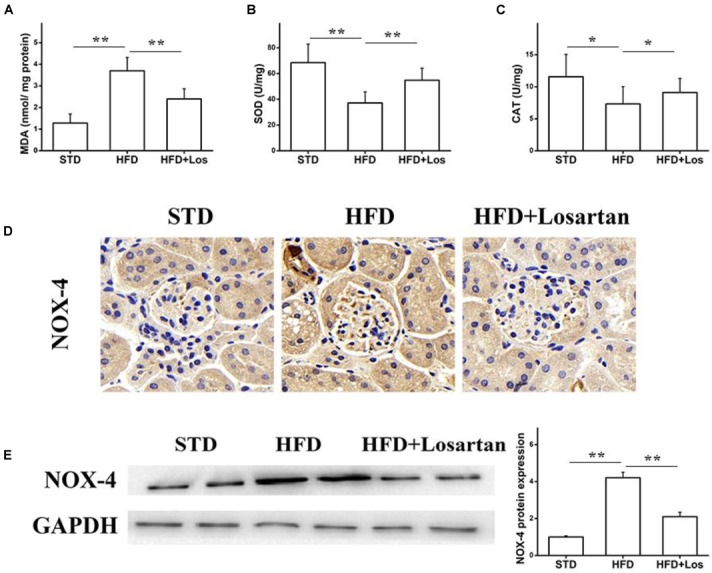
Losartan inhibits oxidative stress damage in kidneys. **(A–C)** The production of lipid peroxidation and related catalyzing enzymes (MDA, SOD, CAT) in all groups. **(D)** Immunohistochemistry analysis of NOX4 in kidneys. **(E,F)** Western blotting assay of NOX4 protein and quantification of NOX4 in different groups. GAPDH was used as a loading control, *n* = 12/group. Three independent experiments were performed. Data are shown as mean ± SD. ^∗^*P* < 0.05, ^∗∗^*P* < 0.01.

Glomerular vascular endothelial cells are increasingly considered to have critical functions in maintaining the glomerular filtration barrier (Sorrentino et al., 2007; Quaggin and Kreidberg, 2008). Oxidative stress is a key factor accounting for endothelial dysfunction in diabetic mice. To assess the effects of losartan on oxidative stress under hyperglycemia, oxidative stress-related markers were examined. The malondialdehyde (MDA) level was increased in kidneys of HFD mice, while losartan treatment decreased this level significantly (Figure 6A). Antioxidants, such as superoxide dismutase (SOD) and catalase (CAT), were also examined. The results showed that the activities of SOD and CAT were decreased in kidneys of HFD mice, while losartan treatment diminished these changes (Figures 6B,C). Immunohistochemistry and Western blotting analyses were carried out to examine the expression of NOX-4. Our data showed that HFD mice had elevated expression of NOX-4 in kidneys compared with STD mice, but losartan treatment markedly reduced NOX-4 expression (Figures 6D,E). These data suggest that losartan functions as a protective agent against oxidative stress damage.

### Losartan Attenuates the TGF-β1/Smad Pathway Under Hyperglycemia

**FIGURE 7:**
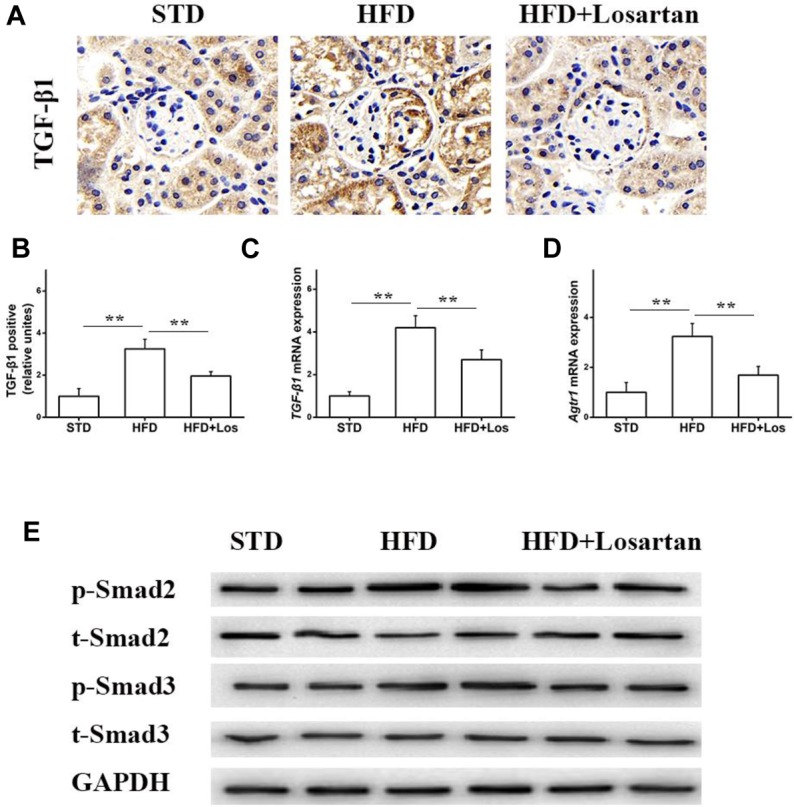
Losartan affects TGF-β1/Smad pathway in kidneys. **(A,B)** Immunohistochemistry analysis for TGF-β1. **(C,D)** The mRNA levels of *TGF-β1* and *Agtr1* in kidneys. **(E)** Protein levels analysis of TGF-β1 and phospho-Smad2/3 proteins from different groups. GAPDH was used as a loading control, *n* = 12/group. Three independent experiments were performed. Data are shown as mean ± SD. ^∗∗^*P* < 0.01.

Previous data have shown that the TGF-β1/Smad pathway is pivotal in development of the EMT (Wang et al., 2017). Therefore, we also examined the status of the TGF-β1/Smad pathway in glomeruli. Immunohistochemistry of TGF-β1 showed that its expression in HFD mice was markedly elevated compared with STD mice, while treatment with losartan decreased TGF-β1 expression under hyperglycemia stress (Figures 7A,B). Real time qPCR results indicated that the mRNA levels of *TGF-β1* and *Atgr1* were increased in HFD compared with STD mice (Figures 7C,D). Western blotting analysis data showed that the protein levels of phospho-Smad2/3 in HFD mice were markedly increased compared with STD mice, but the protein levels of phospho-Smad2/3 in losartan-treated mice were markedly reduced (Figure 7E). These data indicate that losartan suppresses activation of the TGF-β1/Smad2/3 pathway under hyperglycemia stress.

### Losartan Suppresses the HG-Induced EMT *in vitro*

**FIGURE 8:**
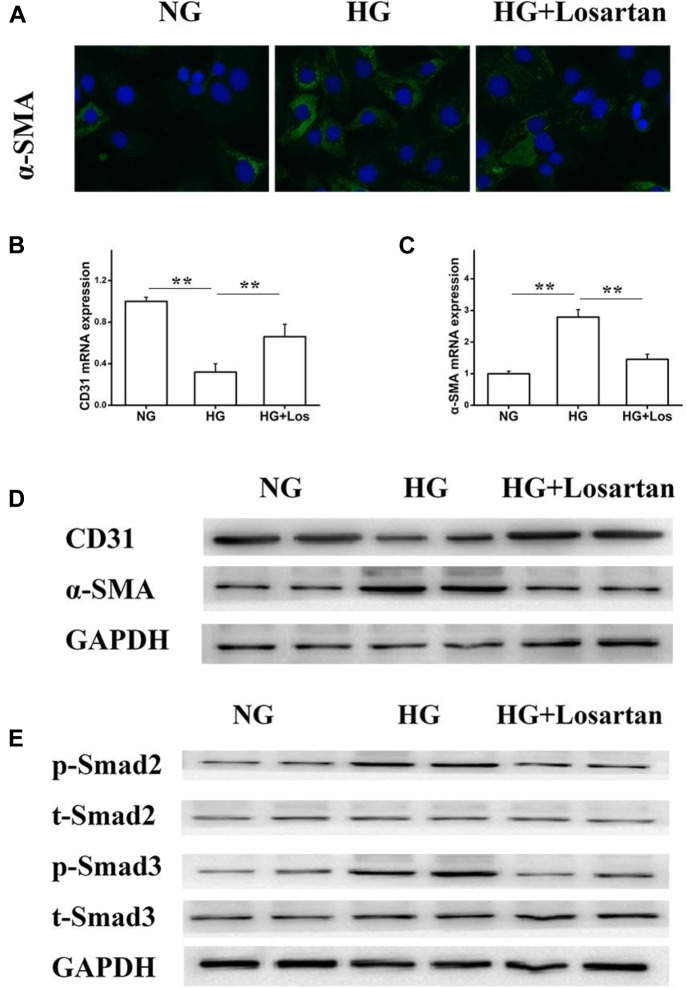
Effect of losartan on high glucose induced endothelial-to-mesenchymal transition in GEnCs. **(A)** Immunofluorescence analysis of α-SMA (fibroblast marker) in different groups. **(B,C)** The mRNA levels of CD31 and α-SMA induced by high glucose in GEnCs. **(D)** Protein levels analysis of CD31 and α-SMA protein induced by high glucose in GEnCs. **(E)** Protein levels analysis of phospho-Smad2/3 proteins induced by high glucose in GEnCs. GAPDH was used as a loading control. Three independent experiments were performed. Data are shown as mean ± SD. ^∗∗^*P* < 0.01.

To further evaluate whether losartan could inhibit the HG-induced EMT *in vitro*, we treated GEnCs with losartan and HG. The immunofluorescence assay showed that the expression of α-SMA in GEnCs was significantly elevated in the HG compared with NG group, while this was reversed by treatment with losartan (Figure 8A). The mRNA level of CD31 was reduced, but the mRNA level of α-SMA was increased in the HG compared with NG group. In contrast, losartan markedly increased the mRNA level of CD31 and decreased the expression of α-SMA (Figures 8B,C). Western blotting analyses indicated that the protein level of CD31 in the HG group was markedly reduced compared with the NG group, while the level of α-SMA in losartan-treated mice was significantly increased (Figure 8D). The protein expression of phospho-Smad2/3 was elevated after exposure to HG when compared with the NG group. By contrast, in the HG + losartan group, the level of phospho-Smad2/3 was significantly reduced (Figure 8E).

**FIGURE 9:**
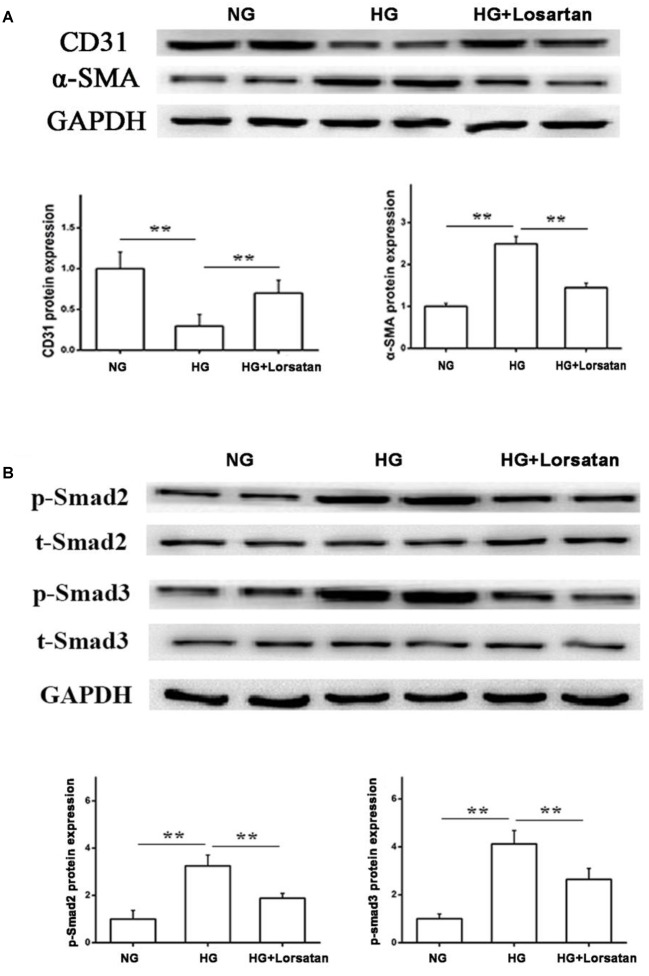
Effect of losartan on high glucose induced endothelial-to-mesenchymal transition in MRGECs. **(A)** Protein levels analysis of CD31 and α-SMA protein induced by high glucose in MRGECs. **(B)** Protein levels analysis of phospho-Smad2/3 proteins induced by high glucose in MRGECs. GAPDH was used as a loading control. Three independent experiments were performed. Data are shown as mean ± SD. ^∗∗^*P* < 0.01.

Furthermore, we isolated the glomerular endothelial cells from mouse kidneys (MRGECs), and then incubated with high glucose with or without losartan. We got the similar results in isolated primary MRGECs (Figures 9A,B). These data indicate that losartan inhibits the EMT progression induced by HG *in vitro*.

## Discussion

High-fat diet -fed mice resembled metabolic syndrome and induces many features of the metabolic syndrome, including IR, hyperlipidaemia, glucose intolerance, increased body weight, adiposity, and hyperinsulinemia (Glastras et al., 2016). T2DM and related kidney disorders are increasing in pandemic proportions largely driven by the exponential rise in obesity (Panwar et al., 2015). An important and growing complication of T2DM is DN (Murphy et al., 2016).

Previous data confirmed that DN is one of the most serious microvascular complications of diabetes. It has various causes including poor glycemic control, high blood pressure, and high cholesterol (especially hypertriglyceridemia) (Ahmad, 2015; Xue et al., 2017). Clinical research shows that DN is responsible for almost half of all incident cases of ESRD in the United States and has become a worldwide health problem (O’Connor and Corcoran, 2012). In addition, the 5-year survival of ESRD patients is less than 40%. The number of DN patients at end stage is still increasing yearly (O’Connor and Corcoran, 2012; Bell et al., 2015). Therefore, it is necessary to find the potential pathogenic mechanism(s) and novel therapeutic agents for treating DN.

It is widely accepted that targeting the renin–angiotensin system in DN by angiotensin receptor blockers provides renal protective effects. However, the potential molecular mechanism behind their protective functions is still incompletely understood (Rahimi, 2016). In this study, we showed that losartan treatment suppressed microalbuminuria, renal hypertrophy, and the EMT, and finally attenuated renal fibrosis, with no impact on the blood glucose level in a HFD-induced hyperglycemia mouse model. Furthermore, losartan inhibited the EMT both *in vivo* and *in vitro*. Simultaneously, losartan inhibited the TGF-β1/Smad pathway and reduced the generation of reactive oxygen species (ROS).

Recent studies found that renal endothelial cells can transform into a fibroblast phenotype in renal dysfunction and fibrosis (Cruz-Solbes and Youker, 2017; Su et al., 2018). The process of cell phenotype reprogramming is called the EMT (Cruz-Solbes and Youker, 2017). The EMT is widely considered as a novel process related to the pathology of renal fibrosis (Cruz-Solbes and Youker, 2017). Importantly, several studies proposed that the EMT contributed to renal fibrosis of DN (Sun et al., 2016). During progression of the EMT, endothelial cells lose endothelial-specific markers such as CD31 and VE-cadherin, while gaining mesenchymal fibroblast-like markers including α-SMA and vimentin (Su et al., 2018). A novel finding of our present study is that losartan administration attenuated the EMT development induced by HG both *in vivo* and *in vitro*.

Decades of research show that the TGF-β1/Smad signaling pathway plays a crucial role in the EMT (Wu et al., 2016; Wang et al., 2017). Inhibition of TGF-β1/Smad signaling can significantly blunt the HG-induced EMT (Zhou et al., 2017). In this research, we found that losartan treatment effectively reversed the increased level of TGF-β1 and the phosphorylation of Smad2/3 in DN. Meanwhile, *in vitro* data indicated that the HG-induced activation of TGF-β1/Smad signaling was markedly suppressed by losartan in GVECs.

It’s widely accepted that ROS have critical functions in the development of DN (Fernandes et al., 2016). Hyperglycemia can induce oxidative stress, which leads to renal cell apoptosis, causing renal injury and kidney fibrosis (Fernandes et al., 2016). In addition, ROS can increase extracellular matrix protein synthesis in renal cells, followed by the development of DN (Aghadavod et al., 2016). MDA is an oxidative stress marker, and increases in CAT and SOD are also considered as markers of oxidative stress (Su et al., 2018). Our results showed that the level of MDA was elevated in the HFD-induced diabetic group, as well as decreased activities of CAT and SOD. However, after losartan treatment, the level of MDA was decreased, and the activities of CAT and SOD were significantly increased. Furthermore, losartan attenuated the renal expression of NOX4, which was induced by hyperglycemia. These data demonstrate that losartan alleviates renal damage and suppresses DN development via inhibition of oxidative stress damage.

## Conclusion

Losartan is a U.S. Food and Drug Administration-approved angiotensin receptor blocker that is used widely to treat hypertension and aortic aneurysm patients (Dietz, 2010). We demonstrated that losartan alleviated renal damage in DN and blunted the EMT in glomeruli. Together, these data suggest that losartan may be developed as a potential agent for the clinical treatment of DN.

## Author Contributions

YL and YY conceived and designed the study. YL, YY, XZ, ZY, LZ, and XL performed experiments. YL, LZ, and XL analyzed data. YL and YY drafted the manuscript. YL, YY, and XL critically revised the manuscript. YL and YY supervised the study. All authors reviewed the manuscript.

## Conflict of Interest Statement

The authors declare that the research was conducted in the absence of any commercial or financial relationships that could be construed as a potential conflict of interest.

## References

[B1] Aghadavod E. Khodadadi S. Baradaran A. Nasri P. Bahmani M. Rafieian-Kopaei M. (2016). Role of oxidative stress and inflammatory factors in diabetic kidney disease. *Iran. J. Kidney Dis.* 10 337–343. 27903991

[B2] Ahmad J. (2015). Management of diabetic nephropathy: recent progress and future perspective. *Diabetes Metab. Syndr.* 9 343–358. 10.1016/j.dsx.2015.02.008 25845297

[B3] Alejandro E. U. Gregg B. Blandino-Rosano M. Cras-Meneur C. Bernal-Mizrachi E. (2015). Natural history of beta-cell adaptation and failure in type 2 diabetes. *Mol. Aspects Med.* 42 19–41. 10.1016/j.mam.2014.12.002 25542976 PMC4404183

[B4] Awazu M. Omori S. Ishikura K. Hida M. Fujita H. (2003). The lack of cyclin kinase inhibitor p27(Kip1) ameliorates progression of diabetic nephropathy. *J. Am. Soc. Nephrol.* 14 699–708. 10.1097/01.ASN.0000051726.41601.C0 12595506

[B5] Bai D. Zhang Y. Shen M. Sun Y. Xia Q. Zhang Y. (2016). Hyperglycemia and hyperlipidemia blunts the Insulin-Inpp5f negative feedback loop in the diabetic heart. *Sci. Rep.* 6:22068. 10.1038/srep22068 26908121 PMC4764951

[B6] Bar-Klein G. Cacheaux L. P. Kamintsky L. Prager O. Weissberg I. Schoknecht K. (2014). Losartan prevents acquired epilepsy via TGF-beta signaling suppression. *Ann. Neurol.* 75 864–875. 10.1002/ana.24147 24659129 PMC4077937

[B7] Barman S. Pradeep S. R. Srinivasan K. (2018). Zinc supplementation alleviates the progression of diabetic nephropathy by inhibiting the overexpression of oxidative-stress-mediated molecular markers in streptozotocin-induced experimental rats. *J. Nutr. Biochem.* 54 113–129. 10.1016/j.jnutbio.2017.11.008 29331868

[B8] Bell S. Fletcher E. H. Brady I. Looker H. C. Levin D. Joss N. (2015). End-stage renal disease and survival in people with diabetes: a national database linkage study. *QJM* 108 127–134. 10.1093/qjmed/hcu170 25140030 PMC4309927

[B9] Bhattacharjee N. Barma S. Konwar N. Dewanjee S. Manna P. (2016). Mechanistic insight of diabetic nephropathy and its pharmacotherapeutic targets: an update. *Eur. J. Pharmacol.* 791 8–24. 10.1016/j.ejphar.2016.08.022 27568833

[B10] Brownlee M. (2005). The pathobiology of diabetic complications: a unifying mechanism. *Diabetes Metab. Res. Rev.* 54 1615–1625. 10.2337/diabetes.54.6.1615 15919781

[B11] Cruz-Solbes A. S. Youker K. (2017). Epithelial to mesenchymal transition (EMT) and endothelial to mesenchymal transition (EndMT): role and implications in kidney fibrosis. *Results. Probl. Cell Differ.* 60 345–372. 10.1007/978-3-319-51436-9_13 28409352

[B12] Daehn I. S. (2018). Glomerular endothelial cells stress and cross-talk with podocytes in the development of diabetic kidney disease. *Front. Med.* 5:76. 10.3389/fmed.2018.00076 29629372 PMC5876248

[B13] Decleves A. E. Rychak J. J. Smith D. J. Sharma K. (2013). Effects of high-fat diet and losartan on renal cortical blood flow using contrast ultrasound imaging. *Am. J. Physiol. Renal Physiol.* 305 F1343–F1351. 10.1152/ajprenal.00326.2013 24049144 PMC3840221

[B14] Dietz H. C. (2010). TGF-beta in the pathogenesis and prevention of disease: a matter of aneurysmic proportions. *J. Clin. Invest.* 120 403–407. 10.1172/JCI42014 20101091 PMC2810090

[B15] Donate-Correa J. Martin-Nunez E. Muros-de-Fuentes M. Mora-Fernandez C. Navarro-Gonzalez J. F. (2015). Inflammatory cytokines in diabetic nephropathy. *J. Diabetes Res.* 2015:948417. 10.1155/2015/948417 25785280 PMC4345080

[B16] Dormandy J. A. Charbonnel B. Eckland D. J. Erdmann E. Massi-Benedetti M. Moules I. K. (2005). Secondary prevention of macrovascular events in patients with type 2 diabetes in the PROactive Study (PROspective pioglitAzone clinical trial in Macrovascular events): a randomised controlled trial. *Lancet* 366 1279–1289. 10.1016/S0140-6736(05)67528-9 16214598

[B17] Fernandes S. M. Cordeiro P. M. Watanabe M. Fonseca C. D. Vattimo M. F. (2016). The role of oxidative stress in streptozotocin-induced diabetic nephropathy in rats. *Arch. Endocrinol. Metab.* 60 443–449. 10.1590/2359-3997000000188 27812607 PMC10118643

[B18] Fu J. Lee K. Chuang P. Y. Liu Z. He J. C. (2015). Glomerular endothelial cell injury and cross talk in diabetic kidney disease. *Am. J. Physiol. Renal Physiol.* 308 F287–F297. 10.1152/ajprenal.00533.2014 25411387 PMC4329492

[B19] Geng J. Yu X. Liu C. Sun C. Guo M. Li Z. (2018). Herba artemisiae capillaris extract prevents the development of streptozotocin-induced diabetic nephropathy of rat. *Evid. Based Complement. Alternat. Med.* 2018:5180165. 10.1155/2018/5180165 29636780 PMC5832121

[B20] Glastras S. J. Chen H. Teh R. McGrath R. T. Chen J. Pollock C. A. (2016). Mouse models of diabetes, obesity and related kidney disease. *PLoS One* 11:e0162131. 10.1371/journal.pone.0162131 27579698 PMC5006968

[B21] Guo F. Sun Y. B. Su L. Li S. Liu Z. F. Li J. (2015). Losartan attenuates paraquat-induced pulmonary fibrosis in rats. *Hum. Exp. Toxicol.* 34 497–505. 10.1177/0960327114543840 25233898

[B22] Kobayashi A. Yamamoto I. Katsumata H. Yamakawa T. Mafune A. Nakada Y. (2015). Change in glomerular volume and its clinicopathological impact after kidney transplantation. *Nephrology* 20(Suppl. 2), 31–35. 10.1111/nep.12463 26031583

[B23] Kolset S. O. Reinholt F. P. Jenssen T. (2012). Diabetic nephropathy and extracellular matrix. *J. Histochem. Cytochem.* 60 976–986. 10.1369/0022155412465073 23103723 PMC3527883

[B24] Komorowsky C. V. Brosius F. C. III Pennathur S. Kretzler M. (2012). Perspectives on systems biology applications in diabetic kidney disease. *J. Cardiovasc. Transl. Res.* 5 491–508. 10.1007/s12265-012-9382-7 22733404 PMC3422674

[B25] Li L. Chen L. Zang J. Tang X. Liu Y. Zhang J. (2015). C3a and C5a receptor antagonists ameliorate endothelial-myofibroblast transition via the Wnt/beta-catenin signaling pathway in diabetic kidney disease. *Metabolism* 64 597–610. 10.1016/j.metabol.2015.01.014 25682062

[B26] Lu Q. Yao Y. Hu Z. Hu C. Song Q. Ye J. (2016). Angiogenic factor AGGF1 activates autophagy with an essential role in therapeutic angiogenesis for heart disease. *PLoS Biol.* 14:e1002529. 10.1371/journal.pbio.1002529 27513923 PMC4981375

[B27] Ma Z. Zhu L. Liu Y. Wang Z. Yang Y. Chen L. (2017). Lovastatin alleviates endothelial-to-mesenchymal transition in glomeruli via suppression of oxidative stress and TGF-beta1 signaling. *Front. Pharmacol.* 8:473. 10.3389/fphar.2017.00473 28769803 PMC5513942

[B28] Mourad A. A. Heeba G. H. Taye A. El-Moselhy M. A. (2013). Comparative study between atorvastatin and losartan on high fat diet-induced type 2 diabetes mellitus in rats. *Fundam. Clin. Pharmacol.* 27 489–497. 10.1111/j.1472-8206.2012.01048.x 22712525

[B29] Murphy D. McCulloch C. E. Lin F. Banerjee T. Bragg-Gresham J. L. Eberhardt M. S. (2016). Trends in prevalence of chronic kidney disease in the united states. *Ann. Intern. Med.* 165 473–481. 10.7326/M16-0273 27479614 PMC5552458

[B30] O’Connor N. R. Corcoran A. M. (2012). End-stage renal disease: symptom management and advance care planning. *Am. Fam. Physician* 85 705–710. 22534347

[B31] Panwar B. Hanks L. J. Tanner R. M. Muntner P. Kramer H. McClellan W. M. (2015). Obesity, metabolic health, and the risk of end-stage renal disease. *Kidney Int.* 87 1216–1222. 10.1038/ki.2014.384 25517912 PMC4449828

[B32] Park C. W. (2014). Diabetic kidney disease: from epidemiology to clinical perspectives. *Diabetes Metab. J.* 38 252–260. 10.4093/dmj.2014.38.4.252 25215271 PMC4160578

[B33] Peng H. Li Y. Wang C. Zhang J. Chen Y. Chen W. (2016). ROCK1 induces endothelial-to-mesenchymal transition in glomeruli to aggravate albuminuria in diabetic nephropathy. *Sci. Rep.* 6:20304. 10.1038/srep20304 26842599 PMC4740844

[B34] Qi H. Casalena G. Shi S. Yu L. Ebefors K. Sun Y. (2017). Glomerular endothelial mitochondrial dysfunction is essential and characteristic of diabetic kidney disease susceptibility. *Diabetes Metab. Res. Rev* 66 763–778. 10.2337/db16-0695 27899487 PMC5319717

[B35] Quaggin S. E. Kreidberg J. A. (2008). Development of the renal glomerulus: good neighbors and good fences. *Development* 135 609–620. 10.1242/dev.001081 18184729

[B36] Rahimi Z. (2016). The role of renin angiotensin aldosterone system genes in diabetic nephropathy. *Can. J. Diabetes* 40 178–183. 10.1016/j.jcjd.2015.08.016 26619914

[B37] Salama Z. A. Sadek A. Abdelhady A. M. Darweesh S. K. Morsy S. A. Esmat G. (2016). Losartan may inhibit the progression of liver fibrosis in chronic HCV patients. *Hepatobiliary Surg. Nutr.* 5 249–255. 10.21037/hbsn.2016.02.06 27275467 PMC4876242

[B38] Satchell S. C. (2012). The glomerular endothelium emerges as a key player in diabetic nephropathy. *Kidney Int.* 82 949–951. 10.1038/ki.2012.258 23064188

[B39] Satchell S. C. Tooke J. E. (2008). What is the mechanism of microalbuminuria in diabetes: a role for the glomerular endothelium? *Diabetologia* 51 714–725. 10.1007/s00125-008-0961-8 18347777 PMC2292427

[B40] Shang J. Zhang Y. Jiang Y. Li Z. Duan Y. Wang L. (2017). NOD2 promotes endothelial-to-mesenchymal transition of glomerular endothelial cells via MEK/ERK signaling pathway in diabetic nephropathy. *Biochem. Biophys. Res. Commun.* 484 435–441. 10.1016/j.bbrc.2017.01.155 28137583

[B41] Sorrentino S. A. Bahlmann F. H. Besler C. Muller M. Schulz S. Kirchhoff N. (2007). Oxidant stress impairs in vivo reendothelialization capacity of endothelial progenitor cells from patients with type 2 diabetes mellitus: restoration by the peroxisome proliferator-activated receptor-gamma agonist rosiglitazone. *Circulation* 116 163–173. 10.1161/CIRCULATIONAHA.106.684381 17592079

[B42] Su Z. Widomski D. Nikkel A. Leys L. Namovic M. Donnelly-Roberts D. (2018). Losartan improves renal function and pathology in obese ZSF-1 rats. *J. Basic Clin. Physiol. Pharmacol.* 29 281–290. 10.1515/jbcpp-2017-0157 29397387

[B43] Sun Y. B. Qu X. Caruana G. Li J. (2016). The origin of renal fibroblasts/myofibroblasts and the signals that trigger fibrosis. *Differentiation* 92 102–107. 10.1016/j.diff.2016.05.008 27262400

[B44] Tsai Y. C. Lee C. S. Chiu Y. W. Lee J. J. Lee S. C. Hsu Y. L. (2018). Angiopoietin-2, renal deterioration, major adverse cardiovascular events and all-cause mortality in patients with diabetic nephropathy. *Kidney Blood Press. Res.* 43 545–554. 10.1159/000488826 29642068

[B45] Wang Z. Han Z. Tao J. Wang J. Liu X. Zhou W. (2017). Role of endothelial-to-mesenchymal transition induced by TGF-beta1 in transplant kidney interstitial fibrosis. *J. Cell Mol. Med.* 21 2359–2369. 10.1111/jcmm.13157 28374926 PMC5618680

[B46] Wei J. Yuan Y. Chen L. Xu Y. Zhang Y. Wang Y. (2018). ER-associated ubiquitin ligase HRD1 programs liver metabolism by targeting multiple metabolic enzymes. *Nat. Commun.* 9:3659. 10.1038/s41467-018-06091-7 30201971 PMC6131148

[B47] Weir G. C. Bonner-Weir S. (2004). Five stages of evolving beta-cell dysfunction during progression to diabetes. *Diabetes Metab. Res. Rev* 53 S16–S21. 10.2337/diabetes.53.suppl_3.S16 15561905

[B48] Wengrower D. Zanninelli G. Latella G. Necozione S. Metanes I. Israeli E. (2012). Losartan reduces trinitrobenzene sulphonic acid-induced colorectal fibrosis in rats. *Can. J. Gastroenterol.* 26 33–39. 10.1155/2012/628268 22288068 PMC3275403

[B49] Wild S. Roglic G. Green A. Sicree R. King H. (2004). Global prevalence of diabetes: estimates for the year 2000 and projections for 2030. *Diabetes Care* 27 1047–1053. 10.2337/diacare.27.5.1047 15111519

[B50] Wu M. Peng Z. Zu C. Ma J. Lu S. Zhong J. (2016). Losartan attenuates myocardial endothelial-to-mesenchymal transition in spontaneous hypertensive rats via inhibiting TGF-beta/Smad signaling. *PLoS One* 11:e0155730. 10.1371/journal.pone.0155730 27176484 PMC4866756

[B51] Wynn T. A. (2008). Cellular and molecular mechanisms of fibrosis. *J. Pathol.* 214 199–210. 10.1002/path.2277 18161745 PMC2693329

[B52] Xu J. Wang J. Cheng Y. Li X. He M. Zhu J. (2018). Glucagon-like peptide-1 mediates the protective effect of the dipeptidyl peptidase IV inhibitor on renal fibrosis via reducing the phenotypic conversion of renal microvascular cells in monocrotaline-treated rats. *BioMed. Res. Int.* 2018:1864107. 10.1155/2018/1864107 29607314 PMC5828432

[B53] Xue R. Gui D. Zheng L. Zhai R. Wang F. Wang N. (2017). Mechanistic insight and management of diabetic nephropathy: recent progress and future perspective. *J. Diabetes Res.* 2017:1839809. 10.1155/2017/1839809 28386567 PMC5366800

[B54] Yao Y. Hu Z. Ye J. Hu C. Song Q. Da X. (2017a). Targeting AGGF1 (angiogenic factor with G patch and FHA domains 1) for blocking neointimal formation after vascular injury. *J. Am. Heart Assoc.* 6:e005889. 10.1161/JAHA.117.005889 28649088 PMC5669188

[B55] Yao Y. Lu Q. Hu Z. Yu Y. Chen Q. Wang Q. K. (2017b). A non-canonical pathway regulates ER stress signaling and blocks ER stress-induced apoptosis and heart failure. *Nat. Commun.* 8:133. 10.1038/s41467-017-00171-w 28743963 PMC5527107

[B56] Ye J. Yao Y. Song Q. Li S. Hu Z. Yu Y. (2016). Up-regulation of miR-95-3p in hepatocellular carcinoma promotes tumorigenesis by targeting p21 expression. *Sci. Rep.* 6:34034. 10.1038/srep34034 27698442 PMC5048429

[B57] Yu C. H. Suriguga S. Gong M. Liu W. J. Cui N. X. Wang Y. (2017). High glucose induced endothelial to mesenchymal transition in human umbilical vein endothelial cell. *Exp. Mol. Pathol.* 102 377–383. 10.1016/j.yexmp.2017.03.007 28347704

[B58] Yu G. Liang X. Xie X. Yang T. Sun M. Zhao S. (2002). Apoptosis, myocardial fibrosis and angiotensin II in the left ventricle of hypertensive rats treated with fosinopril or losartan. *Chin. Med. J.* 115 1287–1291. 12411096

[B59] Zhang T. Yao Y. Wang J. Li Y. He P. Pasupuleti V. (2016). Haploinsufficiency of Klippel-Trenaunay syndrome gene Aggf1 inhibits developmental and pathological angiogenesis by inactivating PI3K and AKT and disrupts vascular integrity by activating VE-cadherin. *Hum. Mol. Genet.* 25 5094–5110. 10.1093/hmg/ddw273 27522498 PMC6078640

[B60] Zhang X. Lian X. Liang D. Zhang L. Liu S. Yang L. (2018). Protective effect of Znt7 on high glucose-induced epithelial-to-mesenchymal transition in renal tubular epithelial cells. *Kidney. Blood Press. Res.* 43 500–512. 10.1159/000488697 29627824

[B61] Zhao L. Zhao J. Wang X. Chen Z. Peng K. Lu X. (2016). Serum response factor induces endothelial-mesenchymal transition in glomerular endothelial cells to aggravate proteinuria in diabetic nephropathy. *Physiol. Genomics* 48 711–718. 10.1152/physiolgenomics.00082.2016 27565710

[B62] Zhou H. T. Yu X. F. Zhou G. M. (2017). Diosgenin inhibits angiotensin II-induced extracellular matrix remodeling in cardiac fibroblasts through regulating the TGFbeta1/Smad3 signaling pathway. *Mol. Med. Rep.* 15 2823–2828. 10.3892/mmr.2017.6280 28260007

